# “And how am I going to ask about this?” – introducing the course “sexual anamnesis” in peer teaching for medical students in Würzburg

**DOI:** 10.3205/zma001592

**Published:** 2023-02-15

**Authors:** Jessica Ruck, Maria Pramberger, Isabelle Späth, Anne Simmenroth, Janina Zirkel

**Affiliations:** 1 Universitätsmedizin Würzburg, Institut für Allgemeinmedizin, Würzburg, Germany; 2 Julius-Maximilians-Universität Würzburg, Medizinische Fakultät, Würzburg, Germany; 3 Julius-Maximilians-Universität Würzburg, Medizinische Fakultät, Lehrklinik, Würzburg, Germany; 4 Universitätsmedizin Würzburg, Medizinische Klinik II, Infektiologie, Würzburg, Germany

**Keywords:** sexual medicine, sexual anamnesis, communicative competence, medical studies, anamnesis

## Abstract

**Aim:**

A course on sexual anamnesis based on peer teaching was developed, piloted, and evaluated at the medical school of the University of Würzburg. The course is part of the expansion of the communication curriculum and in order to close existing gaps in medical education. An implementation of the course in the curriculum is meant to give all students the opportunity to acquire professional skills in this area.

**Method:**

The course consists of knowledge transfer, interactive exercises, role plays with structured feedback, and an exchange with practitioners. A standardized online evaluation of the course took place in regard to teaching quality, subjective learning success, and acceptance. The voluntary course was conducted online in the summer semester of 2021 and in person in the winter semester of 2021/22. A total of 68 students participated. The training of the tutors was realized in cooperation with the “Deutsche Aidshilfe” (DAH).

**Results:**

The course was successfully conducted online and in person. A total of 60 students participated in the evaluation. More than 80% of the students rated the course as structured. They assessed an adequate mix of knowledge transfer and practical exercises. More than half of the students reported that they were more confident in performing sexual anamnesis after they participated in the course. There was an open exchange among the students. More than 90% of the students found the peer teaching by the tutors helpful.

**Conclusion:**

The implementation of the course closes a relevant gap of the curriculum in Würzburg. Sexual anamnesis will be a regular part of the curriculum starting in the winter semester 2022/23. The concept can also be transferred to other universities.

## Zusammenfassung

**Zielsetzung:**

Im Rahmen der Erweiterung des Kommunikationscurriculums und um bestehende Lücken in der medizinischen Ausbildung zu schließen, wurde an der medizinischen Fakultät der Universität Würzburg ein Kurs zur Sexualanamnese im Peer Teaching-Konzept entwickelt, pilotiert und evaluiert. Eine Implementierung des Kurses im Curriculum soll allen Studierenden die Möglichkeit geben, Kompetenzen in diesem Bereich zu erwerben.

**Methoden:**

Der Kurs bestand aus Teilen der Wissensvermittlung, interaktiven Übungen, Rollenspielen mit strukturiertem Feedback und einem Austausch mit behandelnden Ärzt*innen. Das Modul wurde anschließenden mittels standardisierter Online-Evaluation auf Lehrqualität, subjektiven Lernerfolg und Akzeptanz geprüft. Der vorerst freiwillige Kurs wurde im Sommersemester (SoSe) 2021 in einem Online-Format sowie im Wintersemester (WiSe) 2021/22 in Präsenz durchgeführt. Es nahmen insgesamt 68 Studierende teil. Die Schulung der Tutor*innen erfolgte in Kooperation mit der Deutschen Aidshilfe.

**Ergebnisse:**

Sowohl im Online-Format als auch in Präsenz war der Kurs gut durchführbar. Insgesamt 60 Studierende nahmen an der Kursbeurteilung teil. Die Evaluation zeigte zu über 80% eine gute Strukturierung des Kurses mit einer adäquaten Mischung an Wissensvermittlung und praktischen Übungen. Die Teilnahme verhalf über die Hälfte der Studierenden zu mehr Sicherheit in der Durchführung der Sexualanamnese und förderte einen offenen Austausch unter den Studierenden. Von über 90% wurde die Betreuung durch die Tutor*innen als hilfreich empfunden.

**Schlussfolgerungen:**

Durch die Einführung des Kurses wird eine wichtige Lücke im Würzburger Lehrangebot geschlossen. Ab dem Wintersemester 2022/23 wird er fester Bestandteil im Curriculum. Das Konzept könnte auch auf andere Standorte übertragen werden.

## 1. Introduction

Sexuality is directly related to health and well-being of patients [1], [2], [3]. The risk of sexually transmitted diseases together with the prevalence of sexual dysfunctions and associated decreased physical and psychological quality of life are common and relevant issues in the outpatient as well as in the inpatient setting [1], [4]. The well-known human immunodeficiency virus (HIV) can be sexually transmitted. Many patients are still “late presenters” with this virus [5]. Because opportunistic infections cause broadly varying symptoms, patients are presented to different specialist disciplines. For the correct classification of these symptoms, a differentiated sexual anamnesis is important and should therefore also be used interdisciplinary [5], [6], [7], [8]. Especially in the context of primary care, patients often wish to discuss the topic of sexuality with their physician [9], [10], [11]. A Swiss study showed that 90% of the participants would like to be asked about their sexuality by their physician, but only 40% had already been asked about it [9]. The Global Study of Sexual Attitudes and Behaviors also reported that only 9% of the participants from 29 countries had been asked about their sexual health during routine examinations at their family practice [12]. For Germany, this concerned 15% of the women and 18% of the men [11], [12].

Physicians reported a lack of sexual medical knowledge during medical education and training as a reason for their failure to ask the appropriate questions [11], [13]. One of the reasons for insufficient education is that the subject of sexuality has hardly any place in the curricula. The majority of medical faculties in the international context and in Germany as well have established teaching content relating to sexuality, but only ten hours on average are reserved for this [13]. The National Competence-Based Learning Objectives Catalog for Medicine (Nationaler Kompetenzbasierter Lernzielkatalog Medizin, NKLM; [https://www.nklm.de]) in its current version and also in previous versions already lists the “performance of a sexual anamnesis” (14c.2.4.10) as a multidisciplinary learning objective. At the beginning of the NKLM process, when the “Masterplan medicine 2020” was the first concern, it became clear that a longitudinal communication curriculum was urgently needed in Germany. However, the implementation of the communicative competences in the area of sexuality in particular still seems to be deficient. For example, a study of the University Medical Center Hamburg-Eppendorf and the LVR (Landschaftsverbands Rheinland) Clinic Essen showed that students rated the current teaching of sexual medicine as insufficient and that more sexual medicine teaching is required [13].

The Institute of General Medicine and the Skills Lab (Lehrklinik) in Würzburg have many years of experience in teaching communicative skills. However, no module on sexual anamnesis existed so far. Therefore, a sexual anamnesis course was developed to expand existing anamnesis modules and was piloted twice, together with interested female medical students in their fifth year of study, medical staff of the infectious diseases department of the university hospital, and the “Deutsche Aidshilfe” (DAH) (see attachment 1 and attachment 2 ). The course was embedded in a peer teaching concept and was planned with an intensive supervision key. Learning to take medical histories through peer teaching has already been shown to be an effective method [14]. The topic of sexuality is a sensitive subject for students, patients, and medical staff alike. Sometimes, sexuality is a taboo and considered a shameful subject, and many people are hesitant to address the issue. Intensive peer teaching reduces the barriers to talking about sexuality and facilitates an open exchange among students.

## 2. Project description

### 2.1. Students and setting

The course was initially designed as a voluntary course for medical students in the clinical section from the eighth semester. For the pilot course, students in semesters 7 to 12 were included. Participants were recruited through advertising in lectures at the beginning of the semester, through the medical student council, and through the electronic course catalog. The potential dash sample included all students in the clinical section (semesters 6 through 10) with a semester strength of approximately 130 for a total of approximately 650 students. Because of the pandemic, the course was scheduled in two different versions: An online version via the Zoom platform for the summer semester (SuSe) 2021, and a version in person for the winter semester (WiSe) 2021/22.

#### 2.2. Methodological procedure

**Figure 1:**
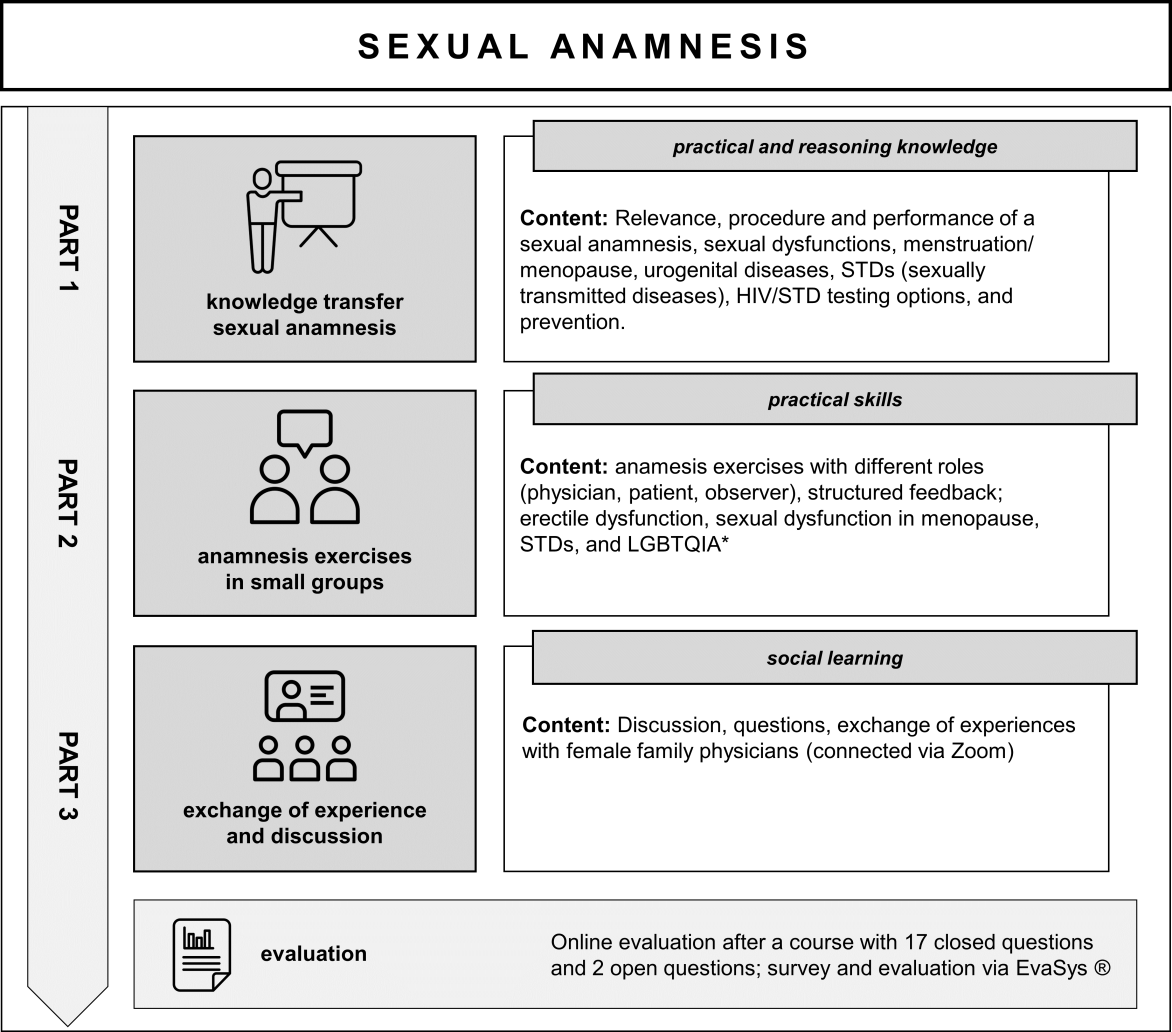
Structure and content topics of the sexual anamnesis course. The illustration was created by the author.

After perceiving the deficit in the area of sexual anamnesis, we first identified in terms of the Kern cycle learning objectives from the NKLM that seemed suitable for the course. Based on this, we developed a three-part course concept with associated materials and teaching methods. First, an introduction was given with a PowerPoint presentation to convey knowledge regarding the content and structure of a sexual anamnesis and common sexual medical problems. Then a practical session was conducted with three role plays, including a feedback evaluation matrix. At the end, open questions were addressed in a discussion with experts from the general medicine or infectiology for anamnesis-related and technical supplements. An overview of the course procedure and content topics is shown in figure 1 (Fig. 1). The online format was prepared and tested using the Zoom platform. Special attention was paid to interactive possibilities such as surveys, whiteboards, and breakout rooms. Professional support and content supervision was provided by docents in general medicine and infectiology. The logistical implementation was organized by DAH-trained tutors (project “let's talk about sex”, [https://www.aidshilfe.de/lets-talk-sex-reloaded]). Additional material for sexual anamnesis for students was provided in the online course room ([https://wuecampus2.uni-wuerzburg.de]) of the University of Würzburg. An evaluation questionnaire was created with the online evaluation tool EvaSys^®^ (see attachment 3 ). To develop the questionnaire, we used and adapted a sample questionnaire that is regularly used to evaluate new teaching units. The evaluation consisted of 17 closed and 2 open questions and was handed out at the end of the seminar. EvaSys^®^ enabled both data collection and a basic evaluation.

#### 2.3. Concept and implementation

The course design included learning objectives at the levels of factual knowledge, practical and reasoning knowledge, and independent practical skills. Based on the NKLM, the following learning objectives were formulated:

- self-reflection of one’s own attitudes toward sexual medicine and sexual anamnesis (see VIII.2-02.1.8)
- acquisition of sexual medical knowledge regarding sexual dysfunctions, menstruation/climacteric period, urogenital diseases, and sexually transmitted diseases (see VI. and VII.)
- acquisition of practical knowledge about taking of a sexual anamnesis and the conduct of conversations on sensitive topics (see VIII.2-02.1.8, VIII.2-03.2.6)
- sensitive, direct, non-judgmental, and trustworthy handling and communication of sexuality, taboo topics, and stigmatized diseases (see VIII.2-02.1.8, VIII.2-03.2.6)
- independent taking of a complete sexual anamnesis (see VIII.2-02.4.7)

Because of the COVID-19 pandemic, the course in the SuSe 2021 took place online. Two identical course were conducted with a total of 22 participants. These were students in their seventh to twelfth semester. Teaching was provided by two docents of infectiology and general medicine and four trained tutors. The course itself was divided into three parts of one hour each.

**Figure 2:**
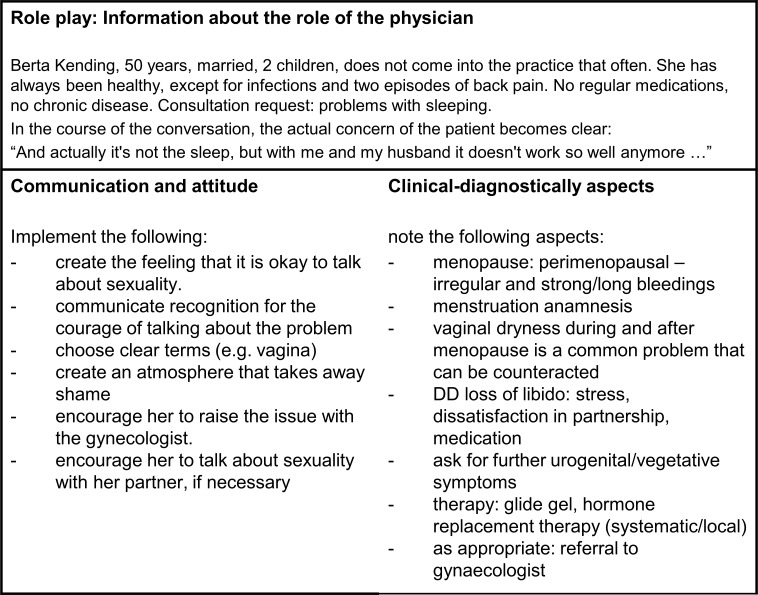
Example of an instruction of the role of the physician. The illustration was created by the author.

**Figure 3:**
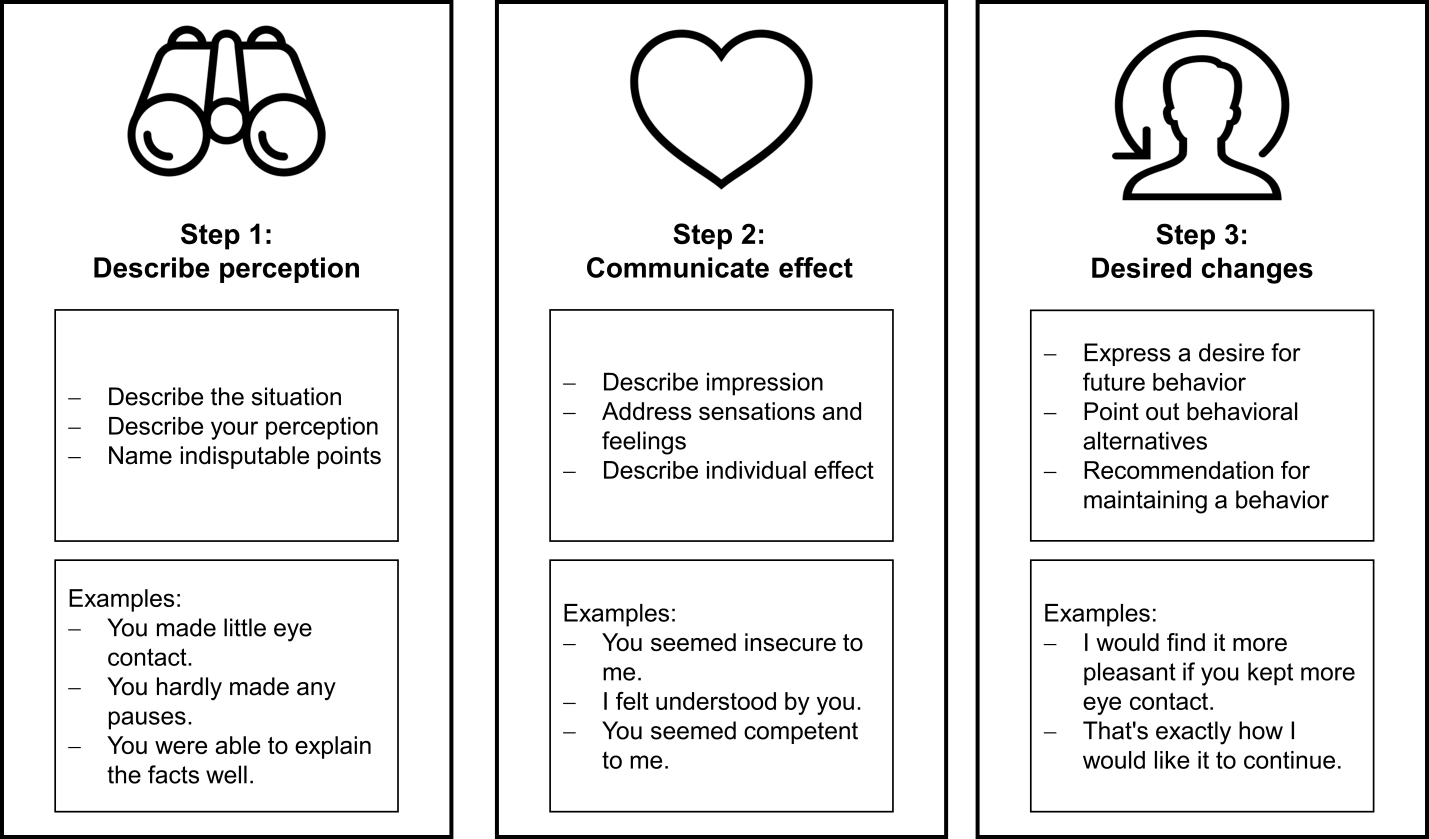
Structured feedback scheme for evaluating the role play for students. The illustration was created by the author.

- In the first part, the docents and tutors gave instructions about important aspects of sexual anamnesis and provided basic information on the occasions of consultation, which were to be practiced in the following role play. A PowerPoint presentation was given, and various applications of Zoom (whiteboards and surveys) were used to make the presentation interactive.
- The second part of the course was focused on anamnesis exercises (AE). Small groups of three students and one tutor each worked in breakout sessions. Three AEs of ten minutes each were conducted by all. An example role instruction is shown in figure 2 (Fig. 2). Each participant took on the role of primary care physician, patient, or observer. Every small group was supervised by a tutor. An AE was followed by a structured feedback session of ten minutes, which was moderated by the supervising tutor. The feedback structure and rules were introduced before, and the observers were instructed to take notes during the AE. The evaluation matrix ensured structured and standardized feedback that could also be supplemented with content-related aspects (see figure 3 (Fig. 3)). The method of AE with structured feedback is already known to the students in Würzburg through anamnesis courses in the preclinical and in the fifth semester.
- In the third part of the course, experiences from the AE were discussed and particular professional questions that could not be answered by the tutors were clarified with the physician and the infectiologist.
- Finally, an evaluation was conducted via a digital questionnaire and brief verbal feedback from participants.

In the WiSe 2021/22, the course was expanded to five evening sessions with a total of 60 participants due to high demand. Forty-six students took part. For this purpose, seven additional tutors were trained in cooperation with the DAH. Since it had become apparent that the students in the SuSe had benefited from their prior knowledge in the subjects of urology, gynecology, general medicine, and especially infectious diseases during the practice discussions, the course was advertised this semester as a “recommended voluntary course from the eighth semester” via the same sources as in the SuSe. The course duration was shortened to 2.5 hours. Evaluations and a thorough faculty debriefing after the SuSe course suggested these changes. The course concept was otherwise retained in its structure from the WiSe. Because it was conducted in person in the WiSe, some adjustments became necessary.

- The first part of the course included the one-hour interactive introduction with a PowerPoint presentation, which was conducted by two female tutors in peer teaching. The interactive elements were replaced by a self-reflection questionnaire, a “world café”, and group work to practice appropriate sexual history questions in specific situations.
- In the second part of the course, the AE (see figure 2 (Fig. 2)) followed by structured feedback (see figure 3 (Fig. 3)) took place in a small-group format and under the supervision of tutors.
- The third part was shortened by 30 minutes compared to the summer term. Students gathered for discussion and exchange of experiences with the docents in the large plenary session. The experts were now docents from the Institute of General Medicine (experienced family physicians), who were connected to the seminar room via zoom.
- The subsequent evaluation of the course took place via smartphone and verbal feedback.

## 3. Results

**Figure 4:**
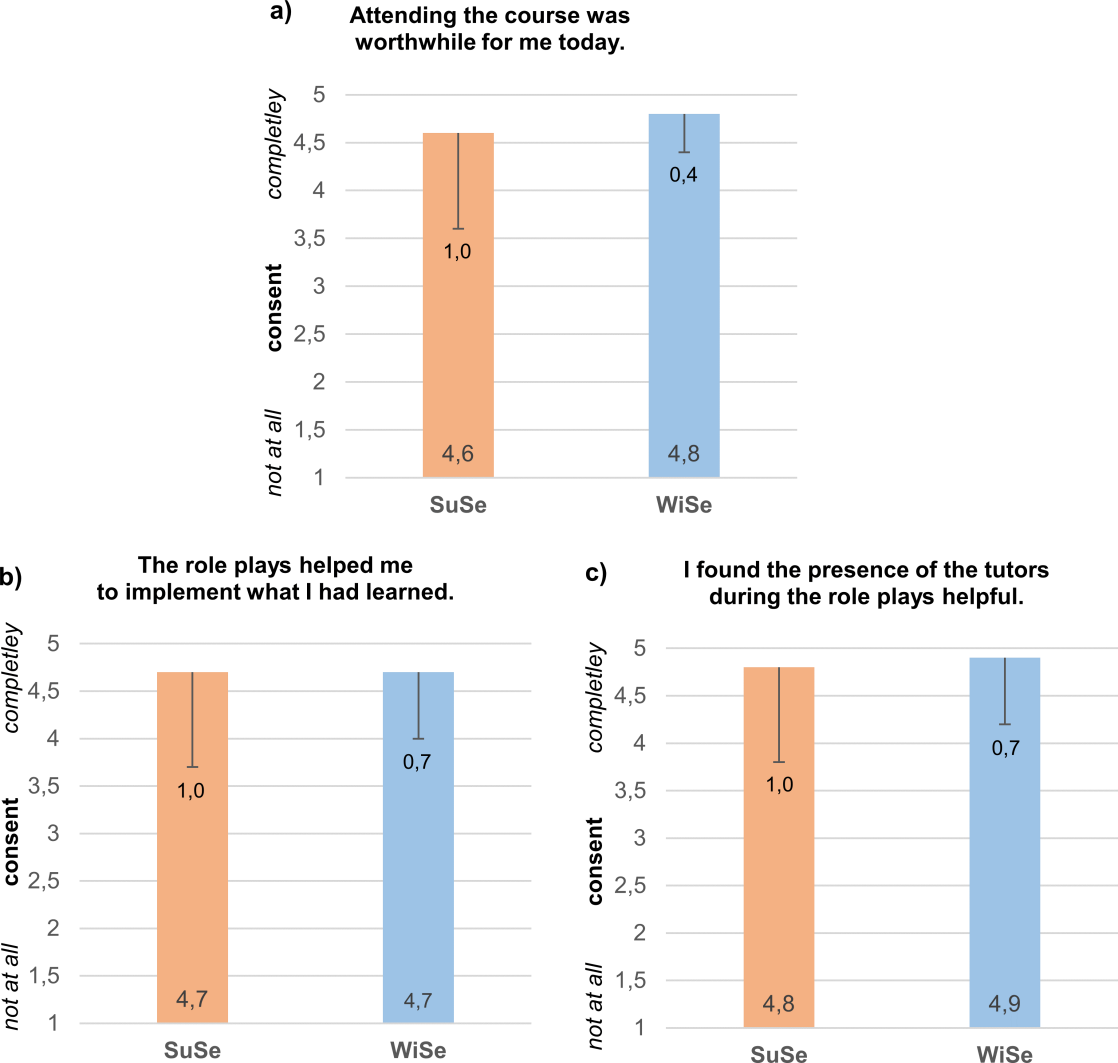
Evaluation results to be highlighted separated by semester (1=I strongly disagree, 5=I strongly agree) with means and standard deviations. The illustration was created by the author.

The courses were evaluated in terms of teaching quality, subjective learning success, student acceptance, and motivation. The evaluation questionnaire was completed by 17 out of 22 students (77%) in the SuSe and 43 out of 46 students (94%) in the WiSe. The evaluation results were consistently positive: Participation in the course was perceived by 11.8% (SoSe) and 16.7% (WiSe) as rather worthwhile and by 82.4% (SuSe) and 83.3% (WiSe) as very worthwhile. This statement showed an agreement of 4.1 (SD=1.0; SuSe) and 4.8 (SD=0.4; WiSe) on average (see figure 4a (Fig. 4)). The AE, which are also a supporting method in existing anamnesis modules of the Würzburg teaching, were a rather helpful possibility for 5.9% (SuSe) and 23.8% (WiSe) and a very helpful possibility for 88.2% (SuSe) and 73.8% (WiSe) to use the previously acquired knowledge in practice. An average of 4.7 (SD=1.0; SuSe) and 4.7 (SD=0.7; WiSe) (see figure 4b (Fig. 4)) agreed that the AE was a good means of transfer support. Peer teaching by tutors was perceived as supportive in particular, with a mean agreement of 4.8 (SD=1.0; SuSe) and 4.9 (SD=0.7; WiSe) (see figure 4c (Fig. 4)). The presence of the tutors was perceived to be helpful by 92.9% (SuSe) and 94.1% (WiSe). The courses were enjoyed for their open atmosphere (88.2% in SuSe and 88.1% in WiSe) and a good mixture of knowledge transfer and practice (81% in SuSe and 88.2% in WiSe). The other results, which can be found in table 1 (Tab. 1), also reflect a successful pilot.

The involvement and intensive support of the tutors, the pleasant working atmosphere, the open and respectful exchange, the clear structure and the well-prepared role plays were reported as positive points in particular in the free-text option of the evaluation. For the upcoming voluntary courses, further information (e.g., handouts) as well as a practice-oriented specialization in culturally and LGBTQIA*-sensitive topics were requested (see table 1 (Tab. 1)).

## 4. Discussion

As part of the expansion of the communication curriculum and to fill existing gaps in medical education, a course on sexual history taking using the peer teaching concept was developed, piloted, evaluated, and finally established in a modified form at the medical school of the University of Würzburg. We were able to follow the Kern cycle throughout here. The evaluation results of the course “taking a sexual anamnesis” showed that students acquired a subjective increase in knowledge and skills and that participation was evaluated as worthwhile and helpful. The course was easily conducted both online and in person and was satisfying from a didactic point of view. Peer teaching in particular was perceived as supportive by participants. Taking a sexual anamnesis requires general communication skills (e.g., active listening and paraphrasing) as well as other specific communication skills related to sexuality (e.g., creating conditions for a conversational atmosphere free from a sense of shame) [15]. The new module as an extension of existing anamnesis modules in the fifth semester is therefore a good addition and can build on the existing basic competences with specific skills. While this course focuses on teaching communicative skills, in other projects at Greifswald University Medical Center, for example, taking a sexual history is embedded in a more holistic, longitudinal concept [4]: The Greifswald curriculum teaches sexual medical basics as well as communicative competences from the perspective of a bio-psycho-social model, considers the relationship-oriented dimension of sexuality, and adds self-awareness aspects. AE in small groups with structured feedback is a suitable and well-tested method for acquiring general and specific communicative skills [2], [16]. The Würzburg course concept included cognitive skills (knowledge transfer by means of a PowerPoint presentation), attitudes (questions for self-reflection by means of a white board, group work, and “world café”) and skills (anamnesis exercises in small groups). Students were thus able to put their acquired knowledge into practice [17], [18]. Peer teaching, as has been widely demonstrated [2], [14], [19], [20], is an effective and popular learning method. Our results also show that peer teaching was found to be particularly helpful. The high tutor ratio (3:1) made it possible to form small groups and offer them intensive supervision. The more intimate setting in the small groups and the low hierarchy to the tutors removed barriers for talking about sexuality. This also facilitated addressing uncomfortable issues or issues that were perceived as in some way shameful. An open and trusting discussion atmosphere was created for the students, which reduced insecurities and facilitated positive experiences. Piloting the course also demonstrates that teaching communicative competences is also possible in an online format. This is confirmed by international studies [21], [22] and is consistent with our experience in digitally teaching alcohol and smoking cessation counseling [23]. In the further development of the course, the demand for a more in-depth exploration of culturally and LGBTQIA* sensitive topics should be addressed. Exploration of sexual identity and sexual orientation is part of one’s sexual history [24]. The increased risk of disease and disadvantage in health care for these patient populations should lead to special general medical consideration [25]. Language barriers, lack of expertise, heteronormativity, and prejudice are just a few of the many difficulties that may be encountered when taking sexual history from these patients [25]. Special sensitivity must be shown here to collect correct information, avoid stigmatization as well as discrimination, and promote a trusting doctor-patient relationship [24]. Appropriate training in these topics is essential in future medical education and training.

### Limitations

The participants were a self-selected sample. Thus, it can be assumed that mainly those students participated who were particularly interested in and open to the topic. In addition, the sample size is small, which limits generalizability. This project was initiated by female students who were particularly motivated and committed. This might have had an influence on the teaching quality or the quality of the peer teaching. Furthermore, there was no objective measurement of learning success, for instance, by an objective structured clinical examination (OSCE), a written exam, a presentation, or similar. The success of the students' acquisition of competences was thus only measured subjectively, which yields only limited information about the actual acquisition of competences.

## 5. Conclusions

The course “taking a sexual anamnesis” was successfully piloted at the Medical School of Würzburg. The course was conducted online and in person and was mainly evaluated positively by the students. The course was again offered in person in the 2022 summer semester for half the students in the semester. Starting in WiSe 2022/23, it will be integrated as a curricular component in the ninth semester. The Würzburg course on taking a sexual anamnesis can serve as an example for other universities to represent sexual medical topics better in the curriculum and to be able to better cope with sexual medical issues in everyday treatment in the long term.

## Note on illustrations

All illustrations were created by the authors themselves. The icons were taken from Icons8 [https://icons8.com/].

## Ethics statement

No data were collected from patients; data collection was anonymous, voluntary, and part of the usual teaching evaluation. Therefore, after consultation with the ethics committee, no application was necessary; a recommended consultation with the data protection officers was carried out and implemented.

## Competing interests

The authors declare that they have no competing interests. 

## Supplementary Material

Voluntary course: sexual anamnesis

Voluntary course SuSe 21: sexual anamnesis

Evaluation sexual anamnesis winter semester 2021/22

## Figures and Tables

**Table 1:**
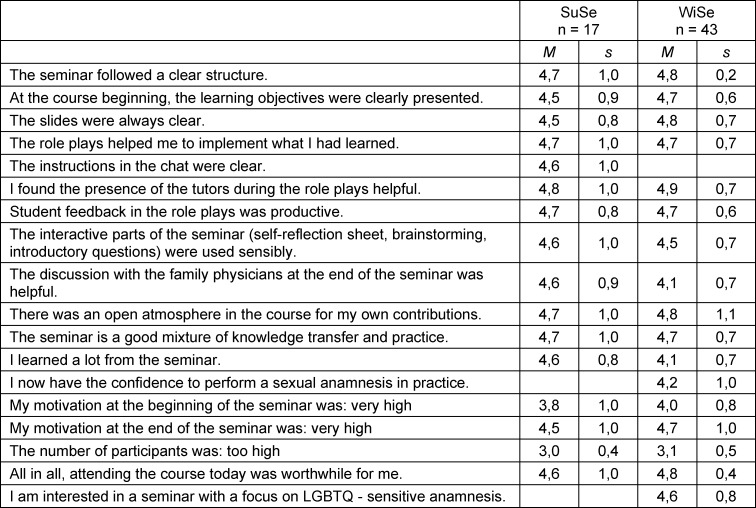
Results of the evaluation separately for summer semester 2021 (SuSe) and winter semester 2021/22 (WiSe) with mean values (Mw) and standard deviations (s); Likert scale: 1=I do not agree at all, 5=I agree completely.
