# Supplementary material for: “And how am I going to ask about this?” – introducing the course “sexual anamnesis” in peer teaching for medical students in Würzburg
Source: GMS J Med Educ. 2023 Feb 15;40(1):Doc10. doi: 10.3205/zma001592 (PMC10010771; doi:10.3205/zma001592)
Supplement: Voluntary course: sexual anamnesis [file JME-40-10-s-001.pdf]

Attachment 1: Voluntary course: sexual anamnesis

Date 1: 25.11.

Date 2: 30.11

Date 3: 2.12

Date 4: 7.12

Date 5: 9.12

respectively 18 – 20 pm in presence (tutor time: 17.45 – 20.15 pm)

Participants: max. 12, from 8./9. semester

**Preperation**	**Responsibilities**
**mal with nformations for partiicipants**	Maria/ Simone
**Zoom link/ technic**	
Preperaton<br>• Folder with materials & participant lists in the tutors' office<br>• 5* Role instructions (for each case for observer, doctor, patient)<br>• Evaluation matrix 5 *<br>• Blueprint<br>• Printed PP<br>• Evaluation QR code<br>• Tutor instructions for moderation of the role plays<br>• Self-reflection sheet for all participants<br>• Zoom - Link for the lecturers absolutely deposit on the Ex_Skills - folder!<br>• Possibly prepare Eddings & Flipcharts/ Answergarden	Isa/ Maria
Before<br>starting PowerPoint<br>• Set up rooms (1* plenary hall with beamer & computer, 3* rooms with tables & 4 - 5 chairs depending on number of trainees)<br>• Prepare list of participants	Isa/ Maria

Time	Dura-tion	Content	Slide	Goals	Methods	Material	who preperates	who refers
18:00	5‘	Check attendance & 3 G's<br>Fill out self-reflection sheet as an offering		Arrive	self-reflection sheets	self-reflection sheets		
	3‘	Welcome<br>Introduction of tutors	1-3	Welcome participants<br>Introduction of the participants and project partners/ how did the course come about?<br>Classification of the course in the curriculum	Vorstellung	Power Point		
	2‘	Structur	4	Outlook on the course of the even-ing	Presentation	Power Point		
	10‘	Brainstorming	5	Align expectations<br>-> Focus of the course: practicing appreciative sexual anamnesis<br>Emphasize relevance of sexual an-amnesis and the course	Interactive: Flip-chart/<br>Answergarden	Flipchart/<br>Answergarden		
	1‘	Why sexual history is important: study.	6-7	There is no "right" sexual anamne-sis, small aids should nevertheless be discussed<br>What should I pay attention to in general during a sexual anamnesis?	Presentation	Power Point		
	3‘	Why talk about sexuality in prac-tice?	8		Presentation	Power Point		
	2‘	What should I look for in a sex-ual history?<br>General aspects	9 - 10	There is no "correct" sexual anam-nesis, nevertheless, small aids are to be discussed<br>What should I pay attention to in general during a sexual anamnesis?	Presentation	Power Point		
	2‘	Structur	11	Organizational framework "Procedure"	Presentation	Power Point		
	2‘	When do I ask about sexuality, possible occasions for a sexual anamnesis	12,13	Short overview for occasions of a sexual anamnesis	Presentation	Power Point		
	15‘	How can I ask about sexuality	14	Practicing / gaining confidence / in-troduction to interview techniques	Interactive: 4 groups with 3 Studierende → 1 issue	Flipchart		

Attachment 1 to Ruck J, Pramberger M, Späth I, Simmenroth A, Zirkel J. *"And how am I going to ask about this?" – introducing the course "sexual anamnesis" in peer teaching for medical students in Würzburg.* GMS J Med Educ. 2023;40(1):Doc10. DOI: 10.3205/zma001592

Time	Dura-tion	Content	Slide	Goals	Methods	Material	who preperates	who refers
	7‘	PPP and HIV-Testing	15,16	Introduce PPP mnemonic, sample sentences, HIV testing	presentation interactive	Power Point		
**18:52**	**8‘**	**Ortswechsel**						
19:00	5´	Practical excersises		Overview of procedure, distribution of tasks, group assignment				
	20´	R1: Blood pressure medication		Exercise, Feedback	role play	Breakout Session		
	20‘	R2: Sleep disorder		Exercise, Feedback	role play	Breakout Session		
	20‘	R3: Inner restlessness		Exercise, Feedback	role play	Breakout Session		
**20:05**	**5‘**	**Change of location**						
20:10	18‘	Plenum: discussion of role plays, time for content-related questions	18	Clarification of open questions	Plenum	Zoom		Dozierende*r
20:28	2‘	Take home messages	19					
20:30	5‘	Goodbye, reflection/ feedback		Conversation<br>QR code		QR Code & Evaluation link		
