# Supplementary material for: “And how am I going to ask about this?” – introducing the course “sexual anamnesis” in peer teaching for medical students in Würzburg
Source: GMS J Med Educ. 2023 Feb 15;40(1):Doc10. doi: 10.3205/zma001592 (PMC10010771; doi:10.3205/zma001592)
Supplement: Voluntary course SuSe 21: sexual anamnesis [file JME-40-10-s-002.pdf]

Attachment 2: Voluntary course SuSe 21: Sexual anamnesis

Uniklinikum Würzburg

Voluntary course SuSe 21:

Sexual anamnesis

Institut of General Medicine
Prof. Dr. A. Simmenroth
medical faculty – skill lab
Dr. Janina Zirkel

Attachment 2 to Ruck J, Pramberger M, Späth I, Simmenroth A, Zirkel J. *"And how am I going to ask about this?" – introducing the course "sexual anamnesis" in peer teaching for medical students in Würzburg.* GMS J Med Educ. 2023;40(1):Doc10. DOI: 10.3205/zma001592

Involvements

Julius-Maximilians-UNIVERSITÄT WÜRZBURG

- Institut of general medicine
- Skill lab
- „Mit Sicherheit Verliebt Würzburg" in cooperation with „Deutschen Aids Hilfe Berlin e.V."

Mit Sicherheit Verliebt Würzburg

LET'S TALK ABOUT SEX

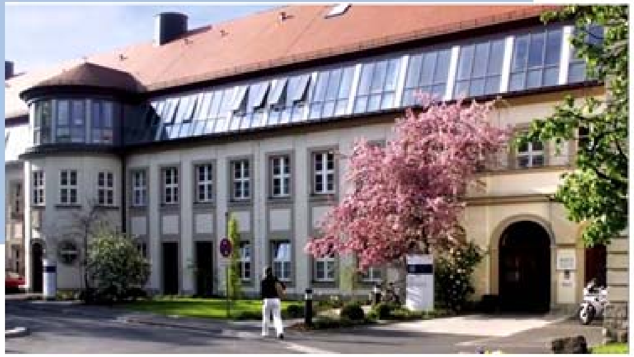

Lehrklinik und Allgemeinmedizin 2 Uniklinikum Würzburg

Attachment 2 to Ruck J, Pramberger M, Späth I, Simmenroth A, Zirkel J. *"And how am I going to ask about this?" – introducing the course "sexual anamnesis" in peer teaching for medical students in Würzburg.* GMS J Med Educ. 2023;40(1):Doc10. DOI: 10.3205/zma001592

Attachment 2: Voluntary course SuSe 21: Sexual anamnesis

current anamnesis (block 1)

What? Where? When, since when? How? How long?
Triggered/aggravated/improved by what? Where from (subjective concept of illness)?

background anamnesis (block 1)

Previous experience with symptoms?
Previous diseases and treatments?
Acute and chronic diseases?
Operations, accidents?
Risk factors (e.g. hypertension, diabetes mellitus)?
Family anamnesis
Allergies? Medications?

psychosocial anamnesis (block 2)

Nationality?
Living situation?
Family context? Social environment?
Job, education?
Existential problems (e.g. debts, unemployment)?

lifestyle/risk behavior (block 2)

nutrition/diet?
Sexuality?
Addictive behavior (e.g. alcohol, nicotine, illegal drugs)?
Health resources, assistive devices?

vegetative anamnesis (block 3)

appetite?
sleep?
digestion?

Attachment 2 to Ruck J, Pramberger M, Späth I, Simmenroth A, Zirkel J. *"And how am I going to ask about this?" – introducing the course "sexual anamnesis" in peer teaching for medical students in Würzburg.* GMS J Med Educ. 2023;40(1):Doc10. DOI: 10.3205/zma001592

Structure today

- Brainstorming
- Why is sexual anamnesis important?
- What do I have to pay attention to?
  - General aspects
  - Structural aspects
- When do I aske for sexuality?
- How can I ask for sexuality?
- Excursus: PPP & STD – Testing
- Practical excercises

Attachment 2 to Ruck J, Pramberger M, Späth I, Simmenroth A, Zirkel J. *"And how am I going to ask about this?" – introducing the course "sexual anamnesis" in peer teaching for medical students in Würzburg.* GMS J Med Educ. 2023;40(1):Doc10. DOI: 10.3205/zma001592

Brainstorming

- ...
- ...
- ...

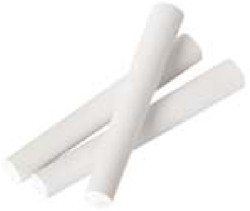

Attachment 2 to Ruck J, Pramberger M, Späth I, Simmenroth A, Zirkel J. *"And how am I going to ask about this?" – introducing the course "sexual anamnesis" in peer teaching for medical students in Würzburg.* GMS J Med Educ. 2023;40(1):Doc10. DOI: 10.3205/zma001592

Why is sexual anamnesis important?

Attachment 2 to Ruck J, Pramberger M, Späth I, Simmenroth A, Zirkel J. *"And how am I going to ask about this?" – introducing the course "sexual anamnesis" in peer teaching for medical students in Würzburg.* GMS J Med Educ. 2023;40(1):Doc10. DOI: 10.3205/zma001592

Patients are open towards talking about sexuality

Male participants answered:	%
It is normal for my doctor to ask me about my sex life in order to give me advice.	95,0
I want to be asked by my doctor about my sex life to get advice on prevention.	90,0
I would be embarrassed.	15,0
The doctor should still ask.	Von den 15%: 76,2
Proportion of patients previously interviewed about your sex life.	40,5

(Meystre-Agustoni et al, 2011, Swiss Medical Weekly)

Attachment 2 to Ruck J, Pramberger M, Späth I, Simmenroth A, Zirkel J. *"And how am I going to ask about this?" – introducing the course "sexual anamnesis" in peer teaching for medical students in Würzburg.* GMS J Med Educ. 2023;40(1):Doc10. DOI: 10.3205/zma001592

Why should sexuality be talked about in practice?

- Sexuality is quality of life
- Professional and complete medical history
- Diseases and medications can affect sexual functions
- Targeted examination and treatment through better information
- Recognition of personal risks
- Opportunity for information, education and prevention

- **OBJECTIVE:** optimal quality of care

Attachment 2 to Ruck J, Pramberger M, Späth I, Simmenroth A, Zirkel J. *"And how am I going to ask about this?" – introducing the course "sexual anamnesis" in peer teaching for medical students in Würzburg.* GMS J Med Educ. 2023;40(1):Doc10. DOI: 10.3205/zma001592

Attachment 2: Voluntary course SuSe 21: Sexual anamnesis

What do I have to pay attention to when taking a sexual anamnesis?

Attachment 2 to Ruck J, Pramberger M, Späth I, Simmenroth A, Zirkel J. *"And how am I going to ask about this?" – introducing the course "sexual anamnesis" in peer teaching for medical students in Würzburg.* GMS J Med Educ. 2023;40(1):Doc10. DOI: 10.3205/zma001592

General aspects of a sexual anamnesis

- Show acceptance and appreciation
- Open and offer space for conversation
- Cultural and religious differences
- LGBTQIA* sensitive language
- Not every problem can or needs to be solved immediately!

- Sexual anamnesis depends always on situation!

Attachment 2 to Ruck J, Pramberger M, Späth I, Simmenroth A, Zirkel J. *"And how am I going to ask about this?" – introducing the course "sexual anamnesis" in peer teaching for medical students in Würzburg.* GMS J Med Educ. 2023;40(1):Doc10. DOI: 10.3205/zma001592

Structur of a sexual anamnesis

- Ask appropriate initial question
- If necessary, point out confidentiality/duty of secrecy
- Clarify terminology used if unclear (e.g. "down below", "intensively stroked")
- In case of language barriers, also use visual material/ model
- PPP- helpful points for an STD anamnesis
- Conclusion

Attachment 2 to Ruck J, Pramberger M, Späth I, Simmenroth A, Zirkel J. *"And how am I going to ask about this?" – introducing the course "sexual anamnesis" in peer teaching for medical students in Würzburg.* GMS J Med Educ. 2023;40(1):Doc10. DOI: 10.3205/zma001592

When do I ask for sexuality?

Attachment 2 to Ruck J, Pramberger M, Späth I, Simmenroth A, Zirkel J. *"And how am I going to ask about this?" – introducing the course "sexual anamnesis" in peer teaching for medical students in Würzburg.* GMS J Med Educ. 2023;40(1):Doc10. DOI: 10.3205/zma001592

Possible occasions for a sexual anamnesis

- Sexual dysfunction
  - Adverse reactions to medications
  - Post OP
  - Psychological/ Stress
  - Age/ menopause
  - Chronic diseases/ new diagnoses
- Menstruation/ menopause
- Urogenital diseases
  - e.g. incontinence
- STDs (= sexual transmitted diseases)
  - Travel history
  - Prevention/ testing services

Attachment 2 to Ruck J, Pramberger M, Späth I, Simmenroth A, Zirkel J. *"And how am I going to ask about this?" – introducing the course "sexual anamnesis" in peer teaching for medical students in Würzburg.* GMS J Med Educ. 2023;40(1):Doc10. DOI: 10.3205/zma001592

Attachment 2: Voluntary course SuSe 21: Sexual anamnesis

How can I ask for sexuality?

Attachment 2 to Ruck J, Pramberger M, Späth I, Simmenroth A, Zirkel J. *"And how am I going to ask about this?" – introducing the course "sexual anamnesis" in peer teaching for medical students in Würzburg.* GMS J Med Educ. 2023;40(1):Doc10. DOI: 10.3205/zma001592

Excursus: PPP

Partner

Practices

Prevention

Attachment 2 to Ruck J, Pramberger M, Späth I, Simmenroth A, Zirkel J. *"And how am I going to ask about this?" – introducing the course "sexual anamnesis" in peer teaching for medical students in Würzburg.* GMS J Med Educ. 2023;40(1):Doc10. DOI: 10.3205/zma001592

HIV/ STD – testing

1. Aids helf/ Checkpoints/ Prevention Projects

2. Health departments: partly free of charge, partly for a fee, anonymous possible

3. Hospital or doctor's office: by name and with note of the result in the patient's file; billing via health insurance company

4. HIV self-test: Pharmacies, drugstores, AIDS help centers.

People from "risk groups" should be tested regularly, irrespective of the occasion.

Attachment 2 to Ruck J, Pramberger M, Späth I, Simmenroth A, Zirkel J. *"And how am I going to ask about this?" – introducing the course "sexual anamnesis" in peer teaching for medical students in Würzburg.* GMS J Med Educ. 2023;40(1):Doc10. DOI: 10.3205/zma001592

Attachment 2: Voluntary course SuSe 21: Sexual anamnesis

Practical Excercises

Attachment 2 to Ruck J, Pramberger M, Späth I, Simmenroth A, Zirkel J. *"And how am I going to ask about this?" – introducing the course "sexual anamnesis" in peer teaching for medical students in Würzburg.* GMS J Med Educ. 2023;40(1):Doc10. DOI: 10.3205/zma001592

Questions and discussion

Attachment 2 to Ruck J, Pramberger M, Späth I, Simmenroth A, Zirkel J. *"And how am I going to ask about this?" – introducing the course "sexual anamnesis" in peer teaching for medical students in Würzburg.* GMS J Med Educ. 2023;40(1):Doc10. DOI: 10.3205/zma001592

Take Home Messages

- Sexual anamnesis is part of every complete anamnesis
- Patient symptoms or problems related to sexuality are more common than physicians think.
- Patients are usually open to a conversation about sexuality (if the situation allows it).
- The "perfect" sexual history does not exist - it is important to open the space for conversations!
- Appreciation and empathy are the basics of every sexual anamnesis.

Attachment 2 to Ruck J, Pramberger M, Späth I, Simmenroth A, Zirkel J. *"And how am I going to ask about this?" – introducing the course "sexual anamnesis" in peer teaching for medical students in Würzburg.* GMS J Med Educ. 2023;40(1):Doc10. DOI: 10.3205/zma001592

Feedback

- Reflecion
- Questions
- Feedback

Attachment 2 to Ruck J, Pramberger M, Späth I, Simmenroth A, Zirkel J. *"And how am I going to ask about this?" – introducing the course "sexual anamnesis" in peer teaching for medical students in Würzburg.* GMS J Med Educ. 2023;40(1):Doc10. DOI: 10.3205/zma001592

Attachment 2: Voluntary course SuSe 21: Sexual anamnesis

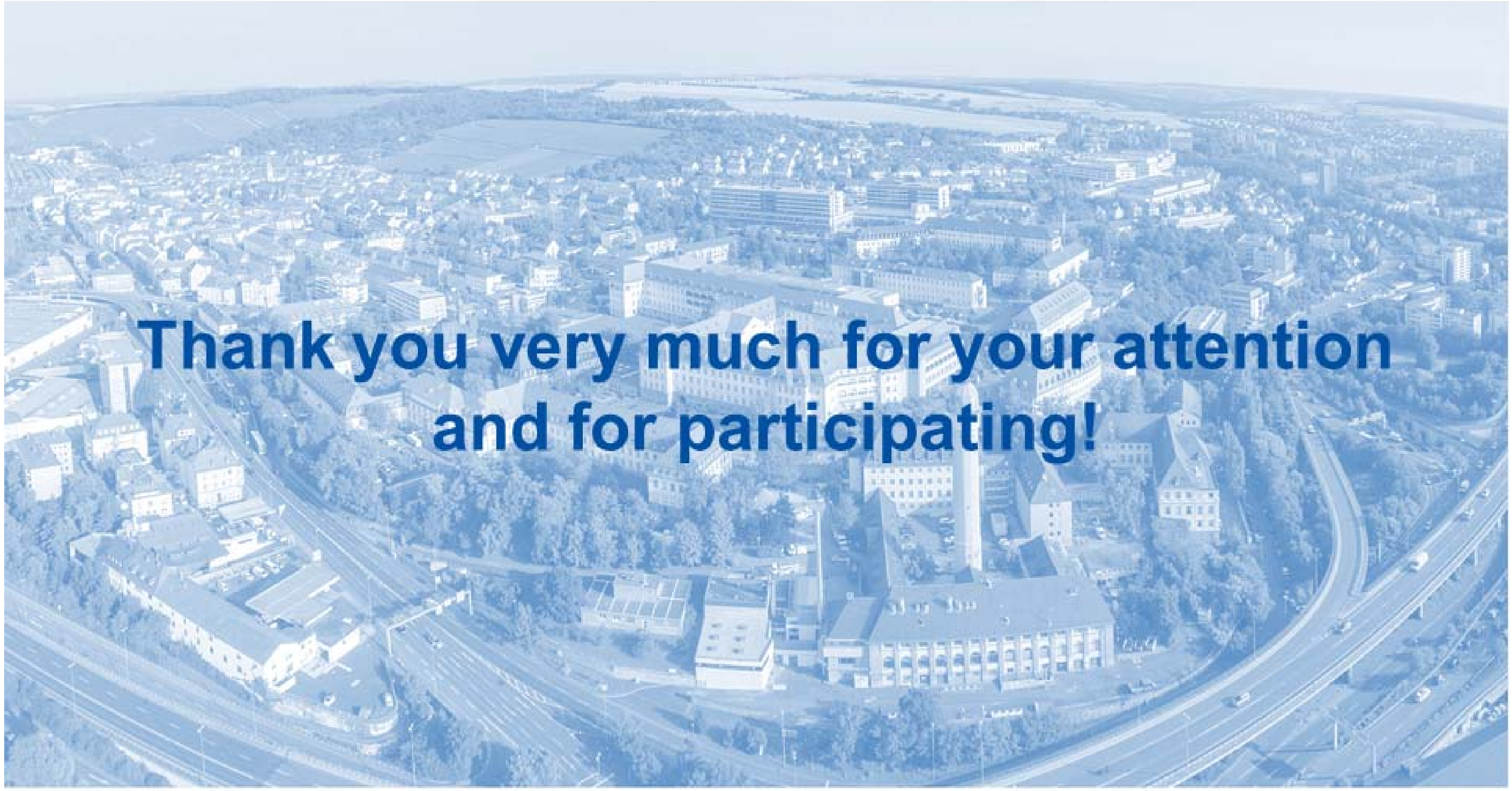

Attachment 2 to Ruck J, Pramberger M, Späth I, Simmenroth A, Zirkel J. *"And how am I going to ask about this?" – introducing the course "sexual anamnesis" in peer teaching for medical students in Würzburg.* GMS J Med Educ. 2023;40(1):Doc10. DOI: 10.3205/zma001592
