# Supplementary material for: “And how am I going to ask about this?” – introducing the course “sexual anamnesis” in peer teaching for medical students in Würzburg
Source: GMS J Med Educ. 2023 Feb 15;40(1):Doc10. doi: 10.3205/zma001592 (PMC10010771; doi:10.3205/zma001592)
Supplement: Evaluation sexual anamnesis winter semester 2021/22 [file JME-40-10-s-003.pdf]

Attachment 3: Evaluation sexual anamnesis winter semester 2021/22

1. demographic data

1.1 age

1.2 gender

1.3 semester

2. content and structure

Scale: 5-point Likert scale

2.1 The seminar followed a clear structure.
2.2 At the beginning of the course, the learning objectives were clearly presented.
2.3 The slides were always clear.
2.4 The role plays helped me to implement what I had learned.
2.5 I found the presence of the tutors during the role plays helpful.
2.6 The feedback from the students in the role plays was productive.
2.7 The interactive parts of the seminar (self-reflection sheet, brainstorming, introductory questions) were used sensibly.
2.8 The discussion with the family physicians at the end of the seminar was helpful.
2.9 There was an open atmosphere in the course for my own contributions.
2.10 The seminar is a good mixture of knowledge transfer and practice.
2.11 I have learned a lot from the seminar in terms of content.
2.12 I now have the confidence to perform a sexual anamnesis in practice.

3. motivation

Scale: 5-point Likert scale

3.1 My motivation at the beginning of the seminar was:
1=very low, 5=very high.
3.2 My motivation at the end of the seminar was:
1=very low, 5=very high
3.3 The number of participants was:
1=too low, 5=too high
3.4 All in all, attending the course today was worthwhile for me:
1=disagree at all, 5=agree completely
3.5 I am interested in a seminar with a focus on LGBTQ - sensitive anamnesis:
1=disagree strongly, 5=agree strongly

4. praise, criticism and feedback

Open questions

4.1 What did you find particularly good about the course?
4.2 Do you have any concrete suggestions for improvement of the seminar?

Attachment 3 to Ruck J, Pramberger M, Späth I, Simmenroth A, Zirkel J. *"And how am I going to ask about this?" – introducing the course "sexual anamnesis" in peer teaching for medical students in Würzburg.* GMS J Med Educ. 2023;40(1):Doc10. DOI: 10.3205/zma001592
